# A Redox-active Mn Porphyrin, MnTnBuOE-2-PyP^5+^, Synergizes with Carboplatin in Treatment of Chemoresistant Ovarian Cell Line

**DOI:** 10.1155/2022/9664636

**Published:** 2022-05-09

**Authors:** Luksana Chaiswing, Chontida Yarana, William St. Clair, Artak Tovmasyan, Ines Batinic-Haberle, Ivan Spasojevic, Daret St. Clair

**Affiliations:** ^1^Department of Toxicology and Cancer Biology, University of Kentucky, Lexington, KY, USA; ^2^Faculty of Medical Technology, Mahidol University, Thailand; ^3^Department of Radiation Oncology, University of Kentucky, Kentucky, USA; ^4^Translational Neuroscience at Barrow Neurological Institute, AZ, USA; ^5^Department of Radiation Oncology, Duke University, USA; ^6^Department of Medicine, Duke University School of Medicine, Durham, NC, USA; ^7^Pharmacokinetics/Pharmacodynamics Core Laboratory, Duke University School of Medicine, Durham, NC, USA

## Abstract

We have employed a redox-active MnP (MnTnBuOE-2-PyP^5+^, Mn(III) meso-tetrakis (N-n-butoxyethylpyridinium-2-yl) porphyrin) frequently identified as superoxide dismutase mimic or BMX-001, to explore the redox status of normal ovarian cell in relation to two ovarian cancer cell lines: OV90 human serous ovarian cancer cell and chemotherapy-resistant OV90 cell (OVCD). We identified that OVCD cells are under oxidative stress due to high hydrogen peroxide (H_2_O_2_) levels and low glutathione peroxidase and thioredoxin 1. Furthermore, OVCD cells have increased glycolysis activity and mitochondrial respiration when compared to immortalized ovarian cells (hTER7) and parental cancer cells (OV90). Our goal was to study how ovarian cell growth depends upon the redox state of the cell; hence, we used MnP (BMX-001), a redox-active MnSOD mimetic, as a molecular tool to alter ovarian cancer redox state. Interestingly, OVCD cells preferentially uptake MnP relative to OV90 cells which led to increased inhibition of cell growth, glycolytic activity, OXPHOS, and ATP, in OVCD cells. These effects were further increased when MnP was combined with carboplatin. The effects were discussed with regard to the elevation in H_2_O_2_ levels, increased oxidative stress, and reduced Nrf2 levels and its downstream targets when cells were exposed to either MnP or MnP/carboplatin. It is significant to emphasize that MnP protects normal ovarian cell line, hTER7, against carboplatin toxicity. Our data demonstrate that the addition of MnP-based redox-active drugs may be used (via increasing excessively the oxidative stress of serous ovarian cancer cells) to improve cancer patients' chemotherapy outcomes, which develop resistance to platinum-based drugs.

## 1. Introduction

The level of prooxidants such as ROS (reactive oxygen species) and RNS (reactive nitrogen species), antioxidant proteins, redox thiol couples, repairment proteins, and metal ions will alter the balance of redox state. This redox imbalance in favor of prooxidants leads to persistent oxidative stress. Several investigators have proposed redox imbalance toward oxidative stress as a mechanism for several cancer features, i.e., increased cell proliferation, resistance to cancer treatment, and epigenetic changes that lead to pathologic and clinical progression of cancers.

Ovarian cancer is female cancer that is considered lethal with the worst prognostic gynecologic malignancy [1, 2]. Based on a recent publication by Siegel et at., it is estimated 19,880 new cases of diagnosed ovarian cancer in 2022, with projected 12,810 death from ovarian cancer, especially in the US [3, 4]. Serous carcinoma is the most common subtype among several subtypes of epithelial ovarian cancer. High-grade serous ovarian carcinoma patients are generally presented at stage III or IV and have a 5-year survival rate of around 49% **[**4, 5**]**. The standard treatment of ovarian cancer is surgery to remove the tumor and subsequent chemotherapy [6]. Despite the advance in diagnosis and treatment, tumor recurrence and chemotherapy resistance remain the significant problems that lead to unsuccessful treatment outcomes [7]. Generally, anticancer drugs currently used for ovarian cancers (including platinum drugs) generate high levels of ROS-mediated oxidative stress [8, 9]. Platinum-related compounds (carboplatin and cisplatin) convert into a highly reactive form upon entering the cell, react rapidly with thiol-containing molecules, and shift cellular redox state to oxidative stress [8, 10]. Li et al. propose that CAMK2G, calcium/calmodulin-dependent protein kinase II gamma, regulates redox homeostasis upon cisplatin treatment by phosphorylating Inositol-Trisphosphate 3-Kinase B at serine 174, which drives cisplatin resistance in ovarian cancer [11]. Further, platinum-related compounds could mediate mitochondrial dysfunction and further augment the production of ROS by inhibiting cytochrome P450 and impairing mitochondrial respiration [12]. The specific mechanism involved in resistant development is still unclear and is being intensively investigated [6, 13–15].

One of the potential mechanisms is “rewiring of the redox state” in which the treatment-resistant cancer cells enhance their antioxidant systems as an adaptive response to overcome higher established ROS levels predominantly generated by mitochondria [16–18]. To combat this adaptive response, several therapeutic approaches have been proposed to push the redox state of the resistant cells towards the extreme oxidizing condition that induces cell death. Those approaches include using low molecular weight redox cycling agents to either increase ROS/RNS formation without interfering with antioxidant systems or sustain ROS/RNS formation but dampen antioxidant defenses [16, 19–23]. Mn-porphyrins have been extensively studied as potent redox-active therapeutics. Initially developed as SOD mimics, Mn-porphyrins exert their protective effects either by direct scavenging of ROS and RNS generated by chemodrugs or by direct interaction with cellular proteins bearing redox-active cysteine [24–27]. Under high oxidizing conditions, in cell lines and pre-clinical animal models, MnPs were shown to suppress growth of glioblastoma, lymphoma, and prostate cancer [16]. In our studies, we have decided to use MnP (MnTnBuOE-2-PyP^5+^), also known as BMX-001, since it has advanced towards clinical trials. It is moderately lipophilic and distributes to high levels in cells and cellular fragments, most so in mitochondria [28]. MnP has been shown to have an excellent safety profile in pre-clinical setting and clinical trials and acted as tumor radio- and chemosensitizer in cellular and animal models. Most MnP studies have demonstrated tumor radiosensitization activity [29–31]. Yet, the molecular mechanism behind its anticancer activity is still not fully understood. The impact of Mn porphyrins on the gynecological cancers in combination with platinum treatments of ovarian cancers has not been explored, especially with relation to platinum resistant in ovarian cancer. Here, we examined if the disparities between the redox state of ovarian cancer versus platinum based-resistant ovarian cancer could improve the treatment efficiency of the resistant phenotypes.

## 2. Methods and Materials

### 2.1. Cells, Reagents, and Treatments

Immortalized human ovarian epithelial cell line hTER7 (HOSE1s-E7/hTERT) is represented a normal ovarian epithelial cell which was kindly provided by Dr. Takeshi Motohara [32]. DMEM/F12 (1: 1 mixture) media were used for culturing of hTER7 cells. The following supplements were used: fetal bovine serum (10%, FBS) (Gemini, West Sacramento, CA), streptomycin (0.1 *μ*g/*μ*l), and penicillin (0.1 units/*μ*l). OV90 human serous ovarian cancer cell lines were purchased from ATCC. Carboplatin-resistant ovarian cancer cell (OVCD) that is derived from OV90 cell line was attained from Dr. Patrick Morin, National Institute on Aging (Baltimore, MD) [33]. The chemoresistant phenotypes of these OV90 variants were described in a prior study and were confirmed with cell viability assay (Supplementary Figure 1). OV90 cells and OVCD cells were cultured in Sigma-Aldrich MCDB 105 mixed with Media 199 (1 : 1 mixture) with 15% FBS, streptomycin (0.1 *μ*g/*μ*l), and penicillin (0.1 units/*μ*l). Cells were cultured in 5% CO_2_ incubator with 37°C temperature. STR (Short Tandem Repeat) analysis, provided by ATCC (Manassas, VA), is used for cell authentication. Most of cell culture reagents were acquired from Thermo Fisher Scientific (Gibco) (Waltham, USA). Unless otherwise specified, the chemicals were brought from Millipore Sigma-Aldrich (St Louis, MO). Chemotherapeutic drugs, cisplatin (CDDP), carboplatin (CBDCA), and paclitaxel (PTX) were obtained from remnant chemotherapy at the Markey Cancer Center's pharmacy. MnP was obtained from Dr. Ines Batinic-Haberle's laboratory (Co-author). Majority of primary antibodies were acquired from Santa Cruz Biotechnology, Inc. Preliminary dose-response experiments with these drugs and time-course were previously tested; specifically, optimum dose and time are used in the manuscript (50 nM MnP, 7 *μ*M CBDCA, 3 *μ*M CDDP, and 5 *μ*M PTX).

### 2.2. Cell Viability Analysis

Colony survival fraction and mitochondrial toxicity based-MTT assay were used for cell viability analysis. The detailed protocols are previously published in Wei et al. [34]. Six-well plates were used for cell seeding; MnP (50 nM) and anticancer drugs were then added. The colonies (contained >50 cells) were visualized with a crystal violet solution after 12 days. In addition, 5 × 10^3^ cells/well (96-well plates) were used for mitochondrial toxicity based-MTT assay. The reduction of purple tetrazolium bromide was colorimetric measured at 570 nm. The synergistic effect of CBDCA and MnP combination treatment (combination index (CI)) is calculated by the CompuSyn 1.0 (CompuSyn).

### 2.3. Production of Intracellular H_2_O_2_

Levels of H_2_O_2_ inside the cells are calculated based on the ability of aminotriazole (3-AT) to inhibit catalase which is detailedly described by Wagner et al. [35]. Briefly, OV90 cells and OVCD cells (between 70 and 90% confluence per 100 mm tissue cultured dish) were treated with 3-AT (20 mM) at 37°C for 0, 5, 10, 15, 30, and 45 min. Cells were harvested and pellets were collected. Levels of intracellular H_2_O_2_ production were measured by a decrease of catalase kinetic rate [36]. Activity of catalase was initiated by adding 30 mM H_2_O_2_ and the disappearance of absorbance at wavelength 240 nm was kinetically observed on a SHIMADZU UV–Vis spectrophotometer.

### 2.4. Extracellular H_2_O_2_ Measurement

External H_2_O_2_ levels are determined with the Amplex Red Peroxidase kit (Thermo Fisher, Waltham, MA). Fifty microliters of the Amplex Red reagents was mixed with the media of ovarian cells. Resorufin, which derived from reaction of H_2_O_2_ with Amplex Red, was measured at an absorbance of 595 nm. H_2_O_2_ standard was used to calculate H_2_O_2_ concentration.

### 2.5. Production of Intracellular ROS/RNS

Detailed experiment can be found in Chaiswing et al. [19]. Briefly, media with H_2_DCFDA (2′,7′-dichlorofluorescein diacetate) or 5-(and-6)-carboxy-2′,7′-dichlorofluorescein diacetate, oxidation-insensitive dye, (CDCFDA) were added to OV90 cells, OVCD cells, or hTER7cells for 30 min and protected from light. To account for H_2_O_2_ production, pretreated cells with polyethylene glycol-catalase (PEG-CAT, 500 units/mL) (Sigma) for 24 h were employed.

### 2.6. Mitochondrial Respiratory and ATP Production

The Agilent Seahorse XFe-96 analyzer (Chicopee, MA, USA) was employed for quantifying mitochondrial respiratory (oxygen consumption rate (OCR)) as well as extracellular acidification rate (ECAR), after 6-hr treatment with MnP or chemodrugs. OV90 cells and OVCD cells (40,000 cells/well) were seeded. OCR and ECAR were measured under the basal condition as previously described by Yarana et al. [37]. Data were presented per protein concentration. Concentrations of ATP (ATP Assay kit, Thermo Fisher) were used to confirm ATP-linked OCR data. Cell homogenates were collected from 10,000 cells and the ATP concentration is calculated based on luciferase producing light against standard ATP.

### 2.7. GSH/GSSG Analyses

Extractions of cell lysates for GSH and GSSG using LC-MS/MS analysis have been previously described in Zhao et al. [38]. To prevent contamination, samples were pelleted at 4000 × g, 10 min. Cell extracts from each cell line and treatment were subjected to pH adjustment (to pH 2.0) before injecting to the instrument.

### 2.8. Western Blots and Thioredoxin 1 (Trx1) Redox Western Blot Analysis

Standard western blot procedure was previously mentioned in Chaiswing et al. [39]. Primary antibodies against glutathione peroxidase (GPx1 and GPx4), Keap1, Nrf2, MnSOD (Upstate Biotech), Trx1 (Ab frontier), *β*-actin, or Tubulin were used. Iodoacetic acid (IAA) (pH 8.3) was used for Trx1 redox western blot to prevent Trx1 oxidation in lysates. MicroSpin G-25 columns (GE Health Care Life Science, Piscataway, NJ) were used to remove unbounded IAA. Trx1 reduced form and Trx1 oxidized form were separated on native polyacrylamide gel. Trx1 bands were scanned and imagined with Li-Core Biosciences Odyssey scanner. The following are Trx1 isoforms: (1) Oxy2, fully oxidized Trx1 two disulfides, (2) R, fully reduced Trx1, and (3) Oxy1, combination of reduced Trx1 with oxidized Trx1, are calculated and present as percentage of total Trx1.

### 2.9. Quantitative Real-Time PCR (qPCR) of Nrf2 and Nrf2's Downstream Targets

MagNA Pure Compact RNA Isolation Kit (LifeScience Roche, Cat# 04802993001) was used for isolation of total RNA from 5 × 10^6^ cells. After reverse-transcribed, first-strand cDNA was preamplified with Transcriptor first-strand cDNA synthesis, Catalog # 04379012001 (Roche) followed by cDNA Pre-Amp Master mix (Cat#06720455001). qPCR are quantified as described in the previous publication Zhao et al. [38]. Expression of 18S was used for normalization.

### 2.10. Intracellular Antioxidants Enzymatic Activity

Cell lysates were used for MnSOD activity gel [40]. NaCN (5 mM) was used to inhibit other SOD activities. MnSOD activity is present at 88 kDa. Intensity of white band on the purple background was measured using the Image J (RD, relative density). The activity of GPxs was measured by the Cayman Chemical GPx activity kit. Catalase activity was quantified after the disappearance of H_2_O_2_ absorbance (at 240 nm).

### 2.11. Liquid Chromatography with Tandem-Mass Spectrometry (LC/MS/MS)

Quantifications of MnP and Carboplatin are adopted and measured on Applied Biosystems/SCIEX API 4000 QTrap MS/MS, Molecular Devices ThermoMax UV/vis plate reader, and a Shimadzu 20A-series HPLC (Duke Cancer Institute PK/PD Core Laboratory), as previously described by Tovmasyan et al. [41]. Cell pellet was mixed with 150 *μ*L water, and the appropriate aliquots of suspension, vortexed prior to each pipetting, were used for carboplatin (C-Pt), MnP, and total protein measurements. Quantification of C-Pt and MnP (LC/MS/MS) and total protein (Bradford assay) data was performed by the Analyst 1.6.2 and SoftMax 1.2.0 software, respectively. A 10 *μ*L of cell suspension (cell pellet +150 *μ*L water) was diluted to the appropriate concentration and used in the standard Bradford assay to obtain the total protein data needed for the normalization of the cell content in the suspension used for C-Pt and MnP LC/MS/MS assays. *Carboplatin (CBDCA)*: In a 500-*μ*L polypropylene vial, 300 *μ*L chloroform, 20 *μ*L of cell pellet/water suspension, 100 *μ*L water, and 10 *μ*L of 1 *μ*g/mL C-Pt-d4 (TRC Canada) were mixed at speed 4, for 40 s Fast-Prep (Thermo-Savant) and centrifuged at room temperature. The upper layer was injected into the instrument. HPLC Column: Agilent Eclipse Plus C18, 1.8 *μ*m, 4.6 × 150 mm with P/N AJ0-4287 Phenomenex, C18 3 × 4 mm. Mobile phase solvents: A -2% acetonitrile, 0.5% formic acid in water; B – acetonitrile; Elution gradient at 1 mL/min: 0-1 min 0-95% B, 1-1.5 min 95% B, 1.5-1.7 min 95-0% B. Run time: 5 min. MRM transitions for C-Pt and C-Pt-d4 (int. std.) (m/z): 372/294 and 376/298, correspondingly. Calibration curves of samples (*n* = 6) in 0–4000 ng/mL range were obtained by adding increasing amounts of C-Pt to water and analyzed alongside study samples. The lower limit of quantification (LLOQ) in the study: 15.6 ng/mL (~12 pmol/mg protein). ***MnP (BMX-001)***: In a 200-*μ*L polypropylene vial, 20 *μ*L of cell pellet/water suspension, 40 *μ*L of 1% formic acid in methanol, and 2 *μ*L of 1.66 *μ*M MnBuOE-d8 (AMRI, NY, USA) were vigorously mixed (Fast-Prep, Thermo-Savant, speed 4, 40 s) and centrifuged at room temperature. A 40 *μ*L aliquot of the sample was mixed with 1% heptafluorobutyric acid (80 *μ*L); 50 *μ*L of mixed liquid was injected into the instrument. Mobile phase solvents, run time, internal standard, and sample calibration were modified from previously published by Tovmasyan et al., to appropriate for OV90 lysates and OVDC lysates [41]. Lower limit of quantification (LLOQ): 50 nM (~15 pmol/mg protein). Samples (*n* = 6) in 0–4000 ng/mL range were obtained by adding increasing amounts of C-Pt to water and were analyzed alongside study samples.

### 2.12. Quantitative and Statistical Data Analyses

At least three separate experiments were employed to validate the results. Data are reported as mean ± standard error. Data analyses were performed with Microsoft Excel or GraphPad Prism. Analyzing the mean difference(s) between the vehicle group and treatment group was employed with Student's *t*-test. Comparing the mean in more than two groups was employed by one-way ANOVA. A post hoc test was subsequently applied. *P* value at ≤.05 was assigned to be significant.

## 3. Results

### 3.1. Carboplatin-Resistant Ovarian Cancer Cell Line Exhibited Oxidized Redox State; High-Level H_2_O_2_ while Low-Level Antioxidants

**Figure 1:**
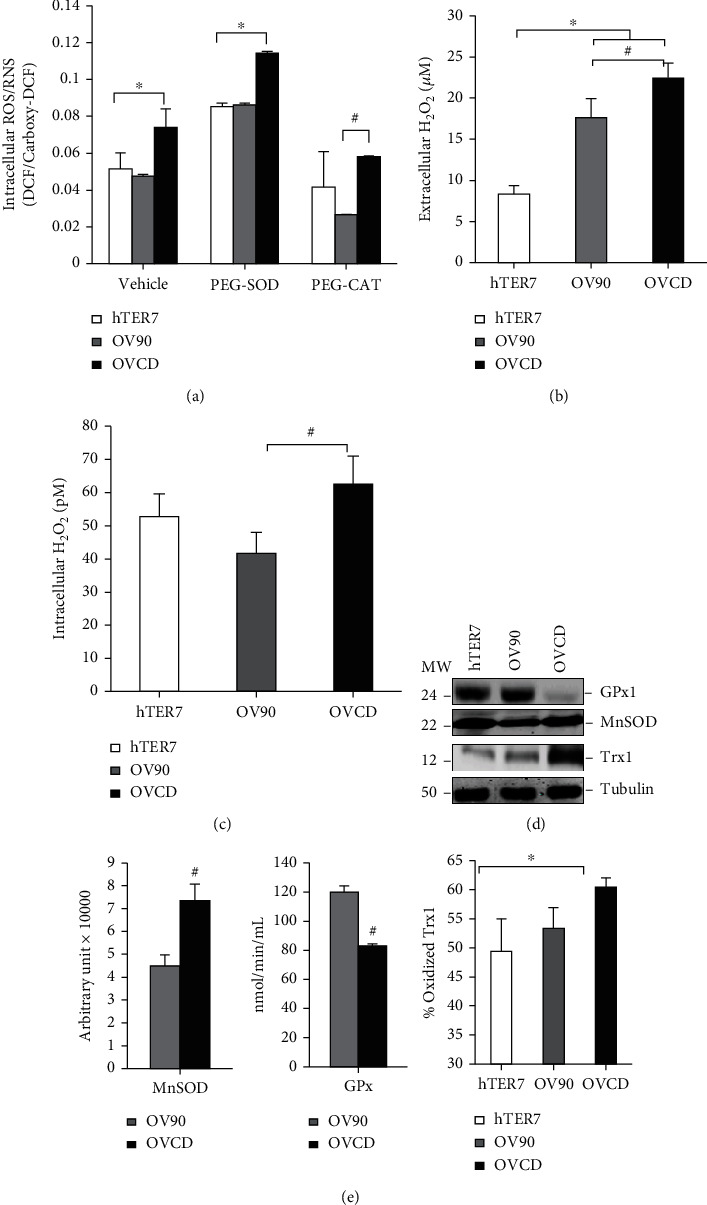
Carboplatin-resistant ovarian cancer exhibited rewired oxidize redox state “high level ROS while low level antioxidants”. (a) Intracellular peroxides levels measured by DCF fluorescence probe. PEG-CAT (500 units) were added 24 hr prior to the assay. (b) Extracellular H_2_O_2_ levels in the media after 24-hr culturing. Amplex Red was used for the assay. (c) Intracellular of H_2_O_2_ in picomole after 24-h culturing. (d) Western blot assay for the steady-state level of antioxidant proteins. Lysates were collected after 24-hr culturing. (e) Antioxidant activities. Cells were collected after 24-hr culturing and sonicated for lysates. MnSOD activity was measured using activity gel with NBT. GPx activity was assessed using a commercial kit. Oxidized form of Trx1 was measured with redox western blot assay. ^∗^*P* ≤ .05 versus hTER7 group. ^#^*P* ≤ .05 versus OV90 group.

To investigate if carboplatin-resistant ovarian cancer cells manifest rewired redox state, we compared the levels of ROS/RNS and antioxidants in carboplatin-resistant ovarian cancer cells (OVCD) to its parental cells (OV90) and noncancerous ovarian surface epithelial cells (hTER7). The result shows that the steady-state intracellular ROS/RNS levels in OV90 and hTER7 cells are similar while they are significantly higher in OVCD. The lower levels of ROS/RNS after treatment with 500 units PEG-CAT suggest that H_2_O_2_ is the major-specific ROS generated **(**Figure 1(a)). Iron fluorescence probe (Phenol Green Fl) demonstrated a higher iron level in OV90 cells and OVCD cells than hTER7 cells, which support the increase of ROS/RNS signal with DCF fluorescence probe (Supplementary Figure 1C) [42]. The concentration of H_2_O_2_ was further measured to confirm the finding. As shown in Figures 1(b) and 1(c), the levels of H_2_O_2_ in OVCD are significantly higher than those in OV90 and hTER7 cells. Interestingly, while extracellular H_2_O_2_ generated by OV90 and OVCD is higher than hTER7 cells; the intracellular H_2_O_2_ level generated by OVCD but not OV90 showed a higher intracellular H_2_O_2_ than hTER7 cells. Expression level of various antioxidants was analyzed by western blotting. OVDC demonstrated the highest protein levels of Trx1 while the lowest level of GPx1 **(**Figure 1(d)). We further confirm the enzyme activities as shown in Figure 1(e). When OVCD and OV90 were compared, the levels of MnSOD and GPx activities were correlated with the protein expression in OVCD, which explains the higher level of H_2_O_2_ in OVCD (Figure 1(d)). Although, Trx1 protein level was significantly increased in OVCD when compared to OV90 (Figure 1(d)), Trx1 is mostly in the oxidized form (Figure 1(e)). Overall, these data support that OVCD cells are in more oxidized redox state.

### 3.2. Increased Mitochondrial and Glycolytic Activities Are Correlated with Carboplatin-Resistant Cancer

**Figure 2:**
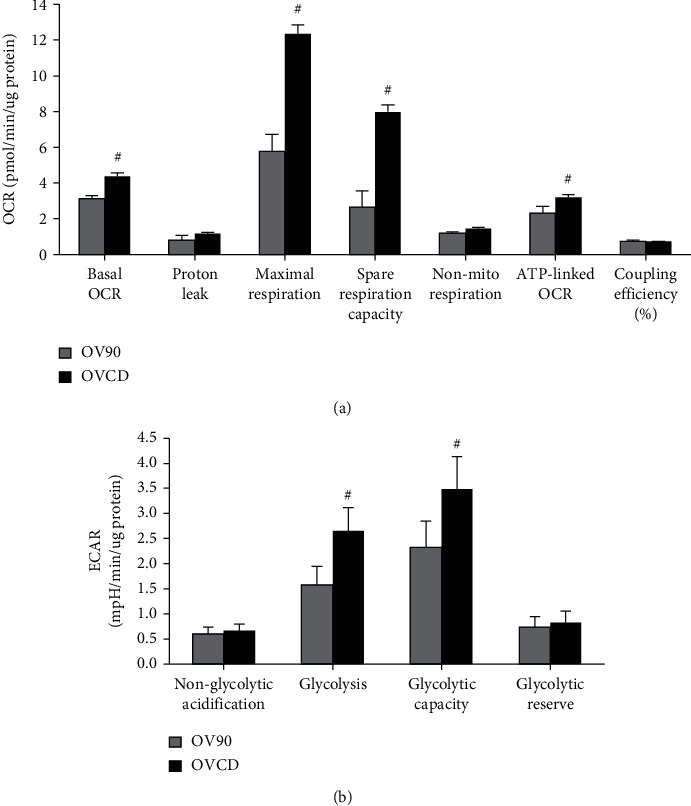
Increased mitochondrial respiratory and glycolytic activity are linked with carboplatin-resistant cancer. (a) Parameters of mitochondrial function calculated from OCR. (b) Glycolytic activity calculated from ECAR of live OV90 cells and OVCD cells. ^#^*P* ≤ .05 versus OV90 group.

Bioenergetic metabolism is critical for cancer progression and aggressiveness. In this study, we compare the level of mitochondrial OXPHOS and glycolysis between OVCD with its parental cell OV90 by measuring OCR and ECAR, respectively. Figures 2(a) and 2(b), show that baseline levels of OCR and ECAR in OVCD were higher than OV90 cells, suggesting that OVCD is metabolically more active than OV90 cells. The increase in OCR in OVCD cell is linked to ATP production. By challenging with FCCP uncoupler, the maximum respiratory and spare respiratory capacity of OVCD were more than two times higher than OV90 cells. Moreover, the glycolytic reserve capacity of OVCD was also higher than OV90 cells. These data suggest that OVCD is superior to OV90 cell with respect to increase in both mitochondrial respiration and glycolysis under the conditions of high energy demand.

### 3.3. MnP Selectively Mediated H_2_O_2_ and Inhibited Growth of Ovarian Cancers but Not Normal Cell

**Figure 3:**
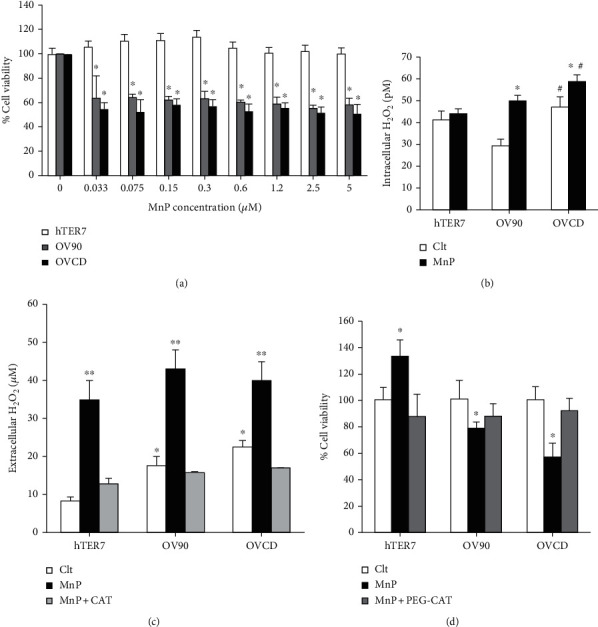
MnP generated H_2_O_2_ and selectively inhibited growth of ovarian cancers but not normal cell. Cells were cultured for 24 hr prior to treatment with 50 nM MnP. (a) Cell viability using MTT assay after 24-hr treatment. % Cell viability was normalized to no-treatment (0 *μ*M). (b) Intracellular of H_2_O_2_ in picomole after 6-h treatment. (c) Extracellular H_2_O_2_ levels in the media after 6-hr treatment. Amplex Red was used for the assay. CAT (1000 units) was added to the media prior to MnP treatment. (d**)** Cell viability using MTT assay after 24-hr MnP treatment. PEG-CAT (500 units) was treated 6 hr prior to MnP treatment. ^∗^*P* ≤ .05 versus hTER7 group. ^∗^*P* ≤ .05 versus non-MnP treatment group. ^#^*P* ≤ .05 versus hTER7 group.

To examine the anticancer efficacy of MnP to chemoresistant ovarian cancer cells versus toxicity to noncancer ovarian epithelial cells, we compare % cell viability of hTER7, OV90, and OVCD in response to MnP (0.033 *μ*M -5 *μ*M). As shown in Figure 3(a), as low as 0.033 *μ*M of MnP induced the cell death of OV90 and OVCD by about 40%. The efficacy of MnP in killing OVCD cell line, at all tested concentrations, was superior to OV90 cell line. However, there was no decrease in cell survival of hTER7 cells at any tested concentration. This result indicates that MnP selectively kills ovarian cancer cells and spares normal ovarian epithelial cell. The data from Figure 1 show that ovarian cancer cells exhibit rewired redox state, as shown by the high levels of intra and extracellular H_2_O_2_. Further, H_2_O_2_ is a signaling ROS that regulates redox-sensitive protein activities that play crucial roles in cell growth and proliferation (at low concentration), as well as cell death (at high concentration) [16]. Therefore, we next investigated if H_2_O_2_ is a crucial molecule by which MnP mediates ovarian cancer cell death. By measuring the intracellular H_2_O_2_, we found that MnP induced a significantly higher amount of H_2_O_2_ in OV90 cell line as well as OVCD cell line, but not in hTER7 cell line when comparing to the non-treated group. Intracellular levels of H_2_O_2_ after MnP treatment in OVCD cell line were significantly greater than in OV90 cell line (Figure 3(b)). Extracellular H_2_O_2_ levels increased to similar levels in all three cell lines with MnP despite a higher baseline in OV90 and OVCD cells when compared to hTER7 cells. The extracellular H_2_O_2_ levels decreased back to baseline with PEG-CAT cotreatment (Figure 3(c)). To investigate if H_2_O_2_ is responsible for the effect of MnP on cell survival, we measured cell viability of hTER7, OV90, and OVCD treated with MnP or combination of MnP and PEG-CAT. As shown in Figure 3(d), MnP alone activates cell growth of hTER7 cells, which was inhibited by PEG-CAT cotreatment (6 hr). In contrast, MnP decreased the cell survival of OV90 to ~80% and decreased cell survival of OVCD to the greater extent of ~60%. However, the cell survival of both OV90 and OVCD cells was rescued by PEG-CAT cotreatment (6 hr). These data imply the prosurvival effect of MnP in normal ovarian epithelial cells but the antitumor effect of MnP in ovarian cancer cells; these effects were mediated by H_2_O_2_. The major source of H_2_O_2_ emerging from MnP treatment is presumably related to the interplay between MnP redox cycling with cellular reductants (most so with ascorbate) and low availability of H_2_O_2_-removing enzymes [41].

### 3.4. Mnp Sensitized Resistant Ovarian Cancer to Carboplatin while Protecting Normal Cells from Chemo-Induced Injury

**Figure 4:**
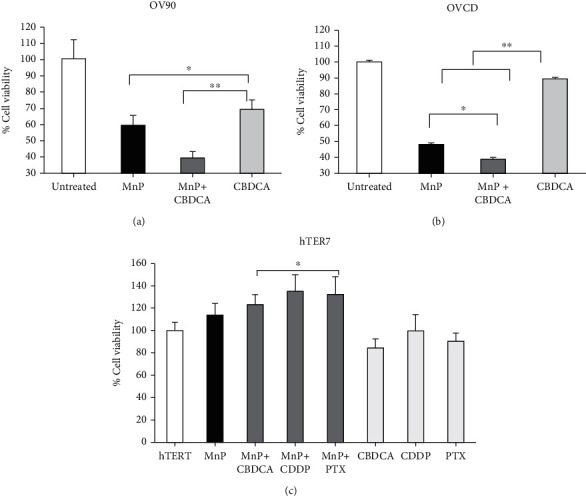
MnP sensitized resistant ovarian cancer to carboplatin while protecting normal cells from chemotherapy-induced injury. Cells were cultured for 24 prior to treatment with 50 nM MnP and/or CBCD (7 *μ*M). (a) OV90 cell viability and (b) OVCD cell viability after treatment of MnP and/or CBCD (24 hr). (c) hTER7 cell viability after treatment of MnP and/or CBCD, CDDP, PTX (24 hr). % Cell viability was normalized to no-treatment. ^∗^*P* ≤ .05 versus hTER7 group. ^∗∗^*P* ≤ .05 versus CBDCA alone.

Since MnP had anticancer effect on OV90 and OVCD, we compare the efficacy of MnP with the first-line antiovarian cancer drugs, carboplatin (CBCDA), and explored the effect of the cotreatment of MnP with carboplatin. As shown in Figure 4(a), MnP alone reduced cell viability of OV90 to about 60%, which was comparable to the cytotoxic effect of carboplatin alone. The combination of MnP and carboplatin treatment reduced cell viability of OV90 further to around 40%. In OVCD, MnP alone decreased cell viability by 50%, the effect was higher than the one of carboplatin alone that caused only 10% reduction in cell viability. Moreover, a combination of MnP and carboplatin treatment further decreased cell viability by 60% (Figure 4(b)). To investigate if MnP prevents the toxicity of antiovarian cancer drugs to normal ovarian epithelial cells, we measured cell viability of hTER7 cells in response to carboplatin, cisplatin (CDDP), or paclitaxel (PTX) with and without MnP cotreatment. The result shows that carboplatin, cisplatin, or paclitaxel treatment decreased cell viability to about 80% and MnP cotreatment prevented cytotoxicity induced by these three anti-cancer drugs (Figure 4(c)). The efficacy of MnP was further tested on ovarian cancer clear cell subtype, TOV21G. Clear cell subtype is an aggressive epithelial ovarian cancer which accounts for <5% of overall ovarian cancers [43]. Although very rare, it has the worst prognosis and is relatively resistant to chemotherapy, especially when tumors recur [44]. Here, we found that 50 nM MnP alone induced TOV21G cell death by 20% (Supplementary Figure 2). Carboplatin at the concentration 3 to 7 *μ*M or cisplatin in the range of 0.5 to 1 *μ*M induced cell death of TOV21G cells at ~50% and 60%, respectively. A combination of MnP with carboplatin or cisplatin showed that MnP synergistically enhanced carboplatin and cisplatin cytotoxicity, which decreased TOV21G cell viability to around 20%.

### 3.5. OVCD Cells Had Greater MnP Accumulation

To compare the accumulation of MnP, carboplatin, or the combination of MnP and carboplatin between OV90 and OVCD, we measured the drug concentration in the cells after one-hour treatment. As shown in Table 1, carboplatin accumulation in OV90 cell line was nearly two times higher than in OVCD cell line, which can be explained by carboplatin-resistant phenotype of OVCD cell line. Interestingly, OVCD cell line took up MnP to a higher level than OV90 cell line. The higher accumulation of MnP did not occur when OVCD cells were treated jointly with MnP/carboplatin. MnP/carboplatin induced higher cell death when compared to MnP or carboplatin alone **(**Figure 4**)**, but the accumulation of MnP, when used jointly with carboplatin, slightly decreased in OV90 and profoundly decreased in OVCD cells.

### 3.6. Cotreatment of MnP and Carboplatin Promotes Production of Cellular Prooxidants

**Figure 5:**
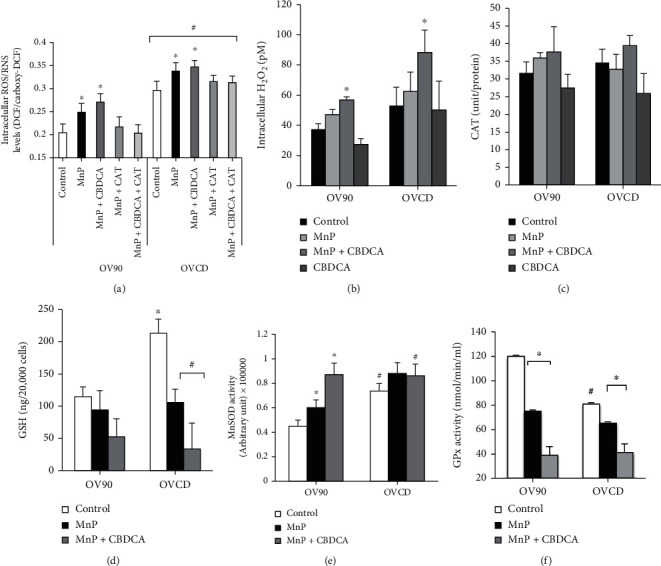
Increasing oxidative stress in cancer by cotreatment of MnP and carboplatin. (a) Intracellular peroxide levels measured by DCF fluorescence probe after 2-hr treatment. PEG-CAT (500 units) were added 24 hr prior to the assay. (b) Intracellular H_2_O_2_ levels after 2-hr treatment. (c) Catalase activity after 2-hr treatment. (d) GSH levels were measured by HPLC after 24-hr treatment. (e) MnSOD activity was measured using activity gel with NBT, after 24-hr treatment. (f) GPx activity was determined using a commercial kit after 24-hr treatment. ^∗^*P* ≤ .05 versus hTER7 group. ^∗^*P* ≤ .05 versus no treatment group. ^#^*P* ≤ .05 versus OV90 group.

We investigated further if MnP or MnP/carboplatin can lead to high H_2_O_2_ production that would cause cell death. By measuring the overall reactive species in OV90 cell line and OVCD cell line, we found that after treatment with MnP or MnP/carboplatin, MnP induced significantly higher levels of ROS/RNS generation comparing to the nontreated control. This is presumably due to the cycling of MnP with cellular reductants, most so ascorbate, which gives rise to the production of superoxide (and its progeny) and subsequently H_2_O_2_ [41] (Figure 5(a)). The combination of MnP with carboplatin slightly but not significantly induced higher ROS/RNS production when compared to MnP alone. The ROS/RNS production from MnP and MnP/carboplatin treatment was blocked by 500 units of PEG-CAT, indicating that the major reactive species was H_2_O_2_. Although intracellular H_2_O_2_ levels in OV90 and OVCD cells were slightly but not significantly increased after MnP treatment, the levels were increased significantly with MnP/carboplatin versus control **(**Figure 5(b)**)**. Interestingly, MnP significantly increased extracellular H_2_O_2_ in OV90 cells and OVCD cells within the first 6 hours of treatment. Combined treatment with MnP and carboplatin further increased extracellular H_2_O_2_. The huge increase in extracellular H_2_O_2_ occurred as early as 5 minutes after MnP treatment and was inhibited by 1000 units catalase (Supplementary Figure 3). The elevation of H_2_O_2_ was correlated with the reduction of GSH level in OVCD by at least 2-fold; comparing to nontreatment control (Figure 5(d)). Yet, the level of GSH in OV90 was slightly but not significantly decreased. However, MnP/carboplatin treatment further decreased GSH in both OV90 cell line and OVCD cell line. We also investigated if MnP and/or carboplatin treatment affected antioxidant enzyme activities in OV90 cells and OVCD cells. The result shows that MnP and/or carboplatin did not have any profound effect on catalase activity in both cell lines (Figure 5(c)). However, MnSOD activity significantly increased after MnP treatment in both OV90 cells and OVCD cells **(**Figure 5(e)**)**. MnP/carboplatin did not further increase MnSOD activity in OVCD cells, only in OV90 cells. This could be explained by the lower accumulation of MnP in OVCD when the cells were treated with both MnP and carboplatin (Table 1). In contrast to MnSOD activity, GPx activity in OV90 cells and OVCD cells was significantly decreased after MnP treatment and further decreased after MnP and carboplatin cotreatment (Figure 5(f)). An elevation of MnSOD activity but a reduction of GPx activity observed after MnP and MnP/carboplatin was consistent with the higher H_2_O_2_ production as well as the lower GSH level.

### 3.7. Cotreatment of MnP and Carboplatin Inhibited Mitochondrial Respiratory and Glycolysis Metabolism in Chemoresistant Ovarian Cancer

**Figure 6:**
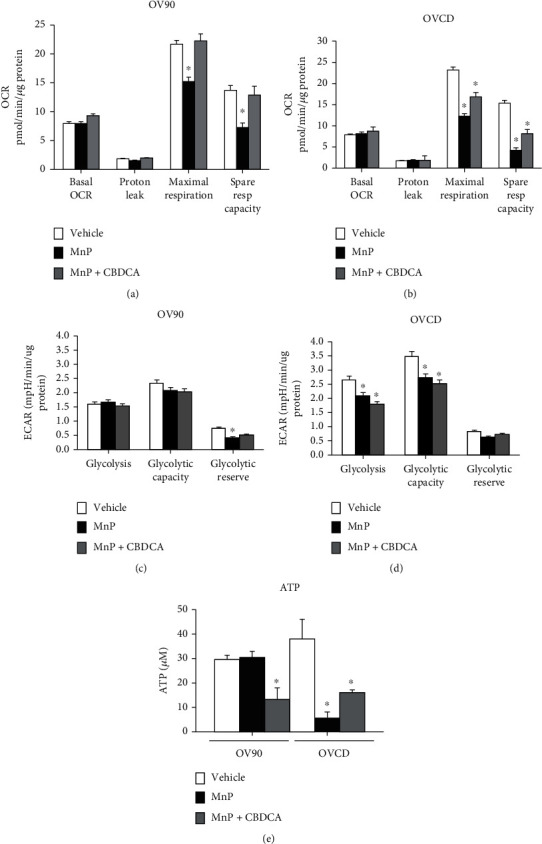
Cotreatment of MnP and carboplatin inhibited mitochondrial respiratory and ATP production in chemoresistant ovarian cancer cells. MnP and/or CBDCA were added to the cells for 6 hr. Live cells were then used for the measurement of OCR and ECAR in OV90 cells (a and c) and in OVCD cells (b and d). (e) Absolute ATP concentration was measured using ATP kits after 24-hr treatment. ^∗^*P* ≤ .05 versus control.

Since mitochondrial and glycolytic activities are correlated with chemoresistance **(**Figure 2**),** we further test if a combination of MnP and carboplatin interferes with cancer metabolism. Mitochondrial respiratory assay and glycolytic stress test were performed 6-hr posttreatments, using a Seahorse Extracellular Flux (XFe-96) instrument. MnP treatment greatly diminished maximum respiration and spare respiratory capacity of both OV90 cell line and OVCD cell line, whereas MnP/carboplatin greatly diminished maximum respiration and spare respiratory capacity only for OVCD cell line **(**Figures 6(a) and 6(b)**).** On the contrary, MnP/carboplatin treatment significantly decreased glycolytic activity and glycolytic capacity in OVCD cells **(**Figures 6(c) and 6(d)**).** The ATP production was measured after 24 hr of treatment, and the data demonstrate that MnP/carboplatin significantly decreased ATP production in both OV90 cells and OVCD cells (Figure 6(e)). Line graphs for Seahorse activity were demonstrated in Supplementary Figure 4. Such data suggest that MnP/carboplatin inhibited ATP production in OV90 cells primarily by targeting mitochondrial metabolism, while in OVCD cells, by targeting both mitochondrial and glycolytic activities.

### 3.8. MnP Inhibited Nrf2 and Nrf2 Downstream Target (Trx1) in Ovarian Cancer Especially Carboplatin-Resistant Cells but Not Normal Cells

**Figure 7:**
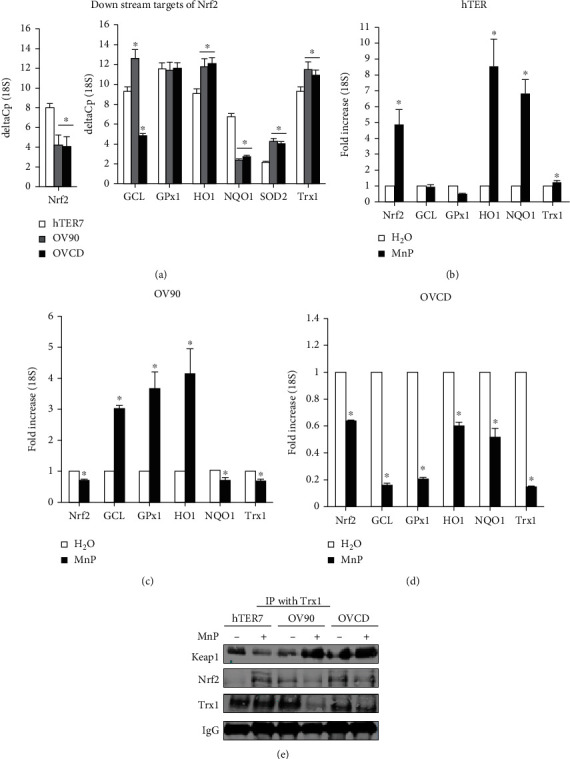
MnP-mediated oxidative stress inhibited Nrf2 and its downstream target, Trx1 in ovarian cancer carboplatin-resistant cell but not in a normal cell. Cells were cultured for 24 hr prior to MnP treatment (6 hr). Cells were then collected, and RNA was isolated for RT-PCR. (a) Nrf2 and its downstream targets were compared between hTER7, OV90, and OVCD cells. Nrf2 and its downstream targets in (b) hTER7 cell line, (c) OV90 cell line, and (d) OVCD cell line, after MnP treatment. (e) Immunoprecipitation (IP) is performed with antibody to Trx1 and western blot analysis with anti-Keap1, Nrf2, and Trx1 antibodies were subsequently performed. ^∗^*P* ≤ .05 when compared to control.

Redox-sensitive transcription factor Nrf2 upregulates several antioxidant enzymes. Since cancer cells rely on antioxidant system for adaptation to high ROS/RNS levels, we first determined Nrf2 mRNA level. As shown in Figure 7(a), mRNA levels of Nrf2 are significantly decreased in OV90 cell line and OVCD cell line when comparing to hTER7 cell line; which correlates with oxidized redox state in OV90 cell line and OVCD cell line. Next, we investigated whether MnP has any effect on Nrf2 and its downstream target in OVCD cells compared to OV90 cells and hTER7 cells. Measurement of Nrf2 target gene expression by RT-PCR shows that MnP significantly induced NAD(P)H Quinone Dehydrogenase 1 (NQO1) and heme oxygenase-1 (HO1) in hTER7 cell line (Figure 7(b)). In contrast, in OV90 cells and OVCD cells, MnP significantly suppressed Nrf2. Furthermore, MnP significantly suppressed Nrf2's downstream targets GCL (glutamate cysteine ligase), GPx1, HO1, NQO1, and Trx1 (Figure 7(d)) in OVCD cells. The effect of MnP on Nrf2 expression was further confirmed with western blot (Supplementary Figure 5). Please note, MnP also suppressed NQO1 and Trx1 in OV90 cells but to a lesser degree compared to OVCD cells (Figure 7(c)). We further investigated the underlying mechanism(s) of MnP differentially affects nonmalignant and malignant ovarian epithelial cells by determining Trx1 interaction with Nrf2 and Keap1 (Kelch-like ECH-associated protein), in the presence and absence of MnP treatment. Immunoprecipitation with Trx1 antibody indicated that MnP promoted the interaction of Trx1 and Nrf2 in hTER7 cells but prevented the interaction of Trx1 and Nrf2 in OV90 cells and OVCD cells (Figure 7(e)). Trx1 regulate Nrf2 by providing electron [45]. The data support the increase in the oxidized state of OV90 cells and OVCD cells upon MnP treatment, potentially via Nrf2 inhibition.

## 4. Discussion

Here in, we demonstrated that MnP has differential antitumor effect on carboplatin-resistant and carboplatin-sensitive ovarian cancer cells. Carboplatin-resistant cells, which acquired rewired redox state manifested by a high level of H_2_O_2_ and a low level of antioxidants including GPx1 and reduced form of Trx1, were more sensitive to MnP than the parental counterpart. Moreover, a combination of MnP with carboplatin increased antitumor effect of MnP. In addition, we also found that MnP protected normal ovarian epithelial cells from chemotherapy-induced cytotoxicity. Our results strengthen the concept of targeting redox dysregulation by MnP as an alternative therapeutic approach for chemotherapy-resistant malignancies and highlight the potential of using tumor redox state to stratify the patients who would have a benefit of MnP treatment.

Prooxidant redox state is associated with tumor aggressiveness and therapy-resistance [16]. This rewired redox state is a weak point of the tumor that can be manipulated by introducing redox-cycling compound that pushes the cells towards extreme oxidative stress and cell death without adverse toxicity to normal cells. Chemoresistant ovarian cancer cells have been shown to be associated with prooxidant redox state and higher metabolism for both mitochondrial and glycolytic pathways. Glycolysis provides intermediates which are required for proteins, lipids, and nucleic acids biosynthesis, as well as rapid ATP production to support the demand of fast-growing cancer cells [46]. Although aerobic glycolysis is extensively active in cancer cells, mitochondrial OXPHOS is still intact, allowing the cells to utilize glutamine for ATP production via Krebs cycle [47]. This metabolic plasticity advocates the cell survival under various nutrient conditions. Fletcher et al. [14] found alterations of specific amino acids in several crucial antioxidant proteins in docetaxel and cisplatin-resistant ovarian cancer cells. Those mutations caused alterations of the enzyme activities that induced cellular prooxidant redox state which is evidenced by a high level of nitrate. However, this study reported a decrease in SOD activity and increases in GPx as well as catalase activities in chemoresistant cells relative to the sensitive cells, which is opposite from our observation. This contradiction suggests the variation of tumor adaptation in response to different chemotherapeutic agents. Despite the difference in antioxidant enzyme activities observed in our study and Fletcher's study, supplementation of SOD increased tumor sensitivity to chemotherapeutic drugs.

MnP is a redox-cycling compound that was initially developed to catalyze superoxide dismutation by accepting an electron from superoxide-producing oxygen followed by generation of H_2_O_2_ as it gives an electron to another molecule of superoxide [48]. Several lines of evidence demonstrated the efficacy of MnP in preventing normal tissue from oxidative stress-induced injury as well as killing cancer cells. In normal tissues, MnP prevents IR-induced bone marrow suppression, erectile dysfunction, pulmonary fibrosis, brain damage, chronic proctitis, and doxorubicin-induced cardiac toxicity [49–51]. Consequently, MnP is in 4 Phase II clinical trials, including glioma, anal cancer, multiple brain metastases, and head and neck cancer. [52]. In preclinical models, MnP mitigates radiation-induced normal tissue damage, including mucositis, xerostomia, and fibrosis, and augments the antitumor effect of radiation [53]. For antitumor efficacy, it was reported by Yulyana et al. that MnP enhanced tumor necrosis factor-related apoptosis-inducing ligand (TRAIL) which then activated glioma cell death [54]. Cytotoxicity of patient-derived glioma cells that are TRAIL-resistant was greatly affected by MnP treatment but not of immortalized normal human astrocytes. The diverse effects observed between glioma cells versus normal astrocytes are further improved with cellular reductants including but not limited to thiols as well as ascorbate [54, 55]. The mitigation ability of MnP when combined with either temozolomide or cisplatin was also accessed and MnP suppressed side effects of temozolomide on mouse rotarod performance [54]. By using murine orthotopic mammary tumor models, Boss et al. recently demonstrated that MnP combination with radiation delays local tumor growth and extends overall survival without promoting pulmonary metastases [56]. The promising data from this and other studies on the effect of MnP on tumor growth and radio and chemoprotection of normal tissue paved way to its progress into clinical trials. These studies are speaking in favor of utilizing MnP for cancer treatment while preserving normal tissue from treatment-related injuries.

Our study further supports this concept with the higher emphasis on the efficacy of MnP in treating chemoresistant ovarian cancers as a single drug or combined with chemotherapy. We explain the impressive efficacy of MnP, when used as a single agent, as follows. MnP accumulates in cancer cell to a high degree and it cycles there with endogenous cellular reductants whereby producing H_2_O_2_. Additionally, we showed, those cancer cell lines, and more so OVCD, have higher levels of H_2_O_2_ than normal cell line but lower GPx levels [57]. In a subsequent step, MnP gets oxidized with intracellular H_2_O_2_ into highly potent oxidizing Mn(V) dioxo species which in turn oxidatively damages proteins [57]. When MnP is combined with carboplatin, the H_2_O_2_ levels are further increased and in turn damage to cellular proteins is increased, which suppresses further cellular viability and as anticipated more so of OVCD cell line than of OV90 cell line [58–61]. Similarly, promoting cytoplasmic H_2_O_2_ production using porphyrin (cationic photosensitizer) in photodynamic therapy is prone to activate cytotoxic activity of human ovarian cancer cell lines such as HeyA8MDR 35026603 and HeyA8 when compared to mouse embryonic fibroblast cell [62]. These studies support the notion that redox-cycling compounds rewire cancer redox state toward oxidizing status which primes cancer cell to death.

Keap1 is a redox-sensitive proximal regulatory protein of Nrf2. Under normal condition, Keap1 ensured low level of Nrf2 by mediating Nrf2 ubiquitination and degradation. Trx1 is a redox-sensitive protein that interacts with other proteins, including Keap1 and Nrf2, and provides electrons through thiol-disulfide exchange to maintain a reduced state and support activities of the proteins [45]. In the cytoplasm, Trx1 interacts and sustains Keap1 in the reduced state preventing nuclear translocation of Nrf2. Nevertheless, Trx1 in the nucleus also interacts and maintains Nrf2 transcriptional activity in nucleus. In normal ovarian epithelial cells, low levels of H_2_O_2_ activate Nrf2 via Keap1 oxidation and simultaneous interaction of Trx1 and Nrf2, leading to the enhancement of Nrf2 transcriptional activity [60, 63]. With regards to the aforementioned studies, recent data from C57BL/6 mice indicate that promoting Trx-1 expression coupled with Nrf2 activation, while decreasing Keap1-overexpression, protects hepatoxicity from tert-butyl hydroperoxide exposure [64]. In contrast, MnP decreased Trx1-Nrf2 interaction while increased Trx1-Keap 1 interaction, in OV90 cells and OVCD cells. Trx1-Keap 1 interaction promoted Nrf2 ubiquitination and decreased Nrf2 translocation to the nucleus. These cascade of events promoted the oxidative stress in the OV90 cells and OVCD cells. When OV90 cells and OVCD cells were compared, Trx1-Nrf2 interactions were similar, but the Nrf2 target genes in OVCD are significantly decreased after MnP treatment. This discrepancy might be due (1) to other antioxidant enzymes that may help maintain redox state in OV90 cells and (2) to the difference in the levels of MnP that entered the cells [65].

Aside from the pharmacodynamics of MnP, the cellular pharmacokinetics of the drug is another factor that contributes to the differential effects of MnP on chemoresistant and chemo-sensitive ovarian cancer cells [61, 65]. In this study, we found ~1.34-fold higher MnP uptake in OVCD when compared to OV90, which suggests the higher accumulation of the drug in more aggressive tumors. Lipophilic MnPs, such as MnTnBuOE-2-PyP^5+^, prefer accumulation in mitochondria [66] and the mitochondrial mass of chemoresistant ovarian cancer cells was reportedly greater than chemo-sensitive cells [67]. Thus, it is possible that mitochondria may contribute to the higher amounts of MnP in aggressive cancers. No information has yet been reported on the effect of chemotherapy on MnP uptake. Our finding in OVCD reveals that carboplatin decreased MnP accumulation by ~1.6 fold. Despite the interference with MnP accumulation, carboplatin increased the antitumor efficacy of MnP, which is speculated to be the result of enhancing Mn redox cycling with additional H_2_O_2_ (originated from carboplatin) which in turn further oxidatively damages cellular proteins.

In conclusion, we showed herein that MnP is highly able to induce cytotoxicity in ovarian OV90 cell line and its resistant analog OVCD cell line at very low nM levels. H_2_O_2_ acts as signaling molecule for inducing cell death when MnP was used as a single drug or when combined with carboplatin. The upregulation of mitochondrial OXPHOS and rewired redox state in ovarian cancer cells could be used as a marker for MnP antitumor efficacy and should be considered for tailoring optimal treatment for the individual cancer patient. Thus, the redox-active MnP-based compounds affect the chemoresistant ovarian cancer cells by upregulating mitochondrial respiration and oxidative stress.

## Figures and Tables

**Table 1:** MnP and carboplatin concentration analysis using LC/MS after 1-hr treatment.

		Concentration (nM; pmol/mL/10^6^ cells)	
Cell	Treatment	MnP	Carboplatin
OV90	MnP	***710.4***	NA
	MnP + carboplatin	690.7	5444.74
	Carboplatin	NA	4959.57

OVCD	MnP	***950.1***	NA
	MnP + carboplatin	600.1	2884.10
	Carboplatin	NA	2673.85

## Data Availability

Data available on request.

## Supplementary Materials

Supplementary Materials Supplementary Figure 1: testing chemotherapy resistant in OVCD cells and OV90 cells. Supplementary Figure 2: synergistic effect of MnP and platinum compounds on TOV 21G. Supplementary Figure 3: cell viability correlated with H_2_O_2_ production after MnP and CBCDA treatment at various time points. Supplementary Figure 4: cotreatment of MnP and carboplatin inhibited mitochondrial respiratory and ATP production in chemoresistant ovarian cancer. Supplementary Figure 5: western blots demonstrated expression of Nrf2 protein after MnP treatment 6 hr.
