# Supplementary material for: A Redox-active Mn Porphyrin, MnTnBuOE-2-PyP5+, Synergizes with Carboplatin in Treatment of Chemoresistant Ovarian Cell Line
Source: Oxid Med Cell Longev. 2022 May 9;2022:9664636. doi: 10.1155/2022/9664636 (PMC9313984; doi:10.1155/2022/9664636)
Supplement: Supplementary Materials — Supplementary Figure 1: testing chemotherapy resistant in OVCD cells and OV90 cells. Supplementary Figure 2: synergistic effect of MnP and platinum compounds on TOV 21G. Supplementary Figure 3: cell viability correlated with H2O2 production after MnP and CBCDA treatment at various time points. Supplementary Figure 4: cotreatment of MnP and carboplatin inhibited mitochondrial respiratory and ATP production in chemoresistant ovarian cancer. Supplementary Figure 5: western blots demonstrated expression of Nrf2 protein after MnP treatment 6 hr. [file 9664636.f1.pdf]

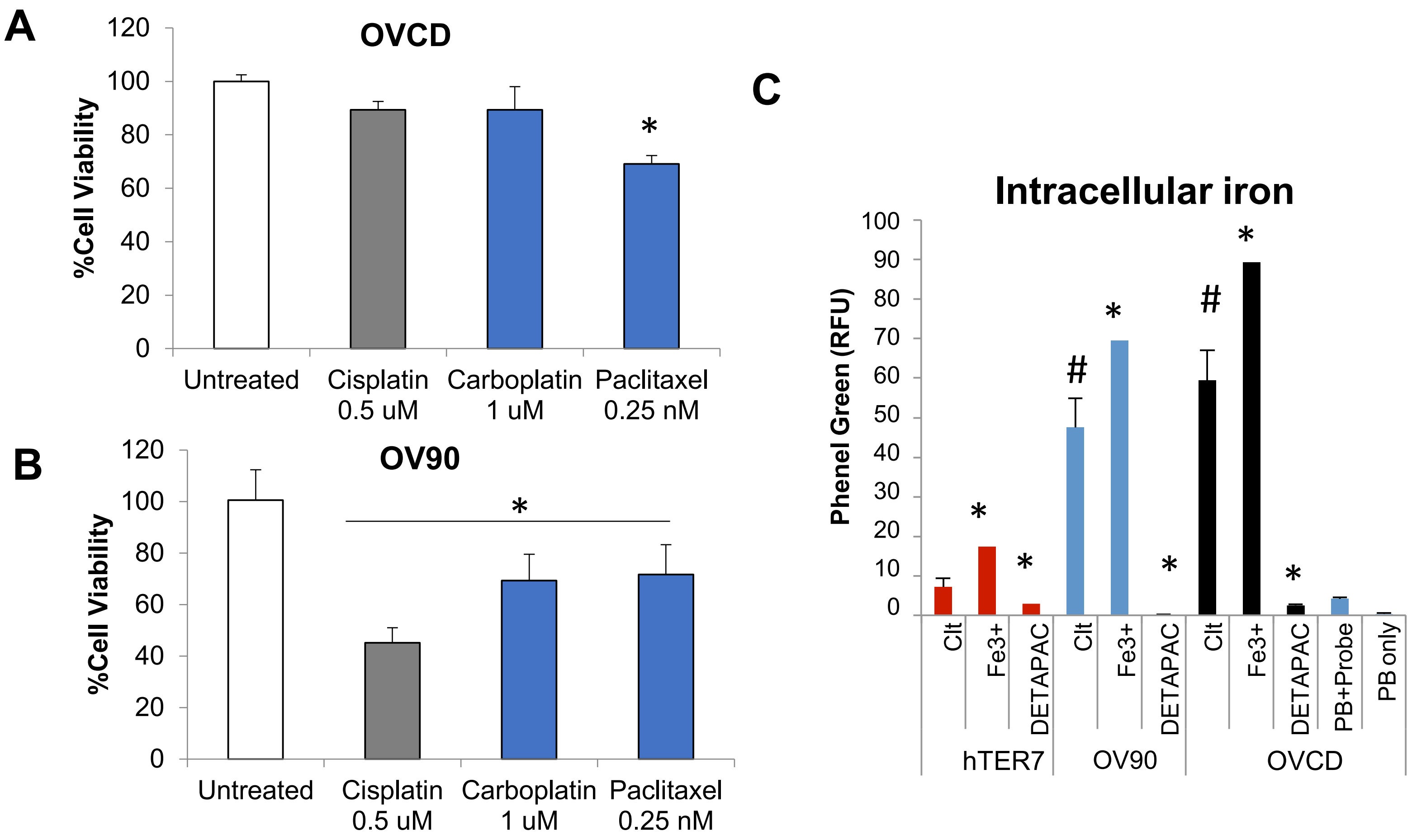

Supplementary Figure 1. Testing chemotherapy resistant in **(A)** OVCD cells and **(B)** OV90 cells. Cells were cultured for 24 prior to treatment. Cell viability using colony survival assay was performed. % Cell viability was normalized to untreated group. **(C)** Intracellular iron was measured using Phenel Green fluorescence probe. *P-value ≤ 0.05 when compared to untreated. #P-Value≤0.05 when compared to hTER7.

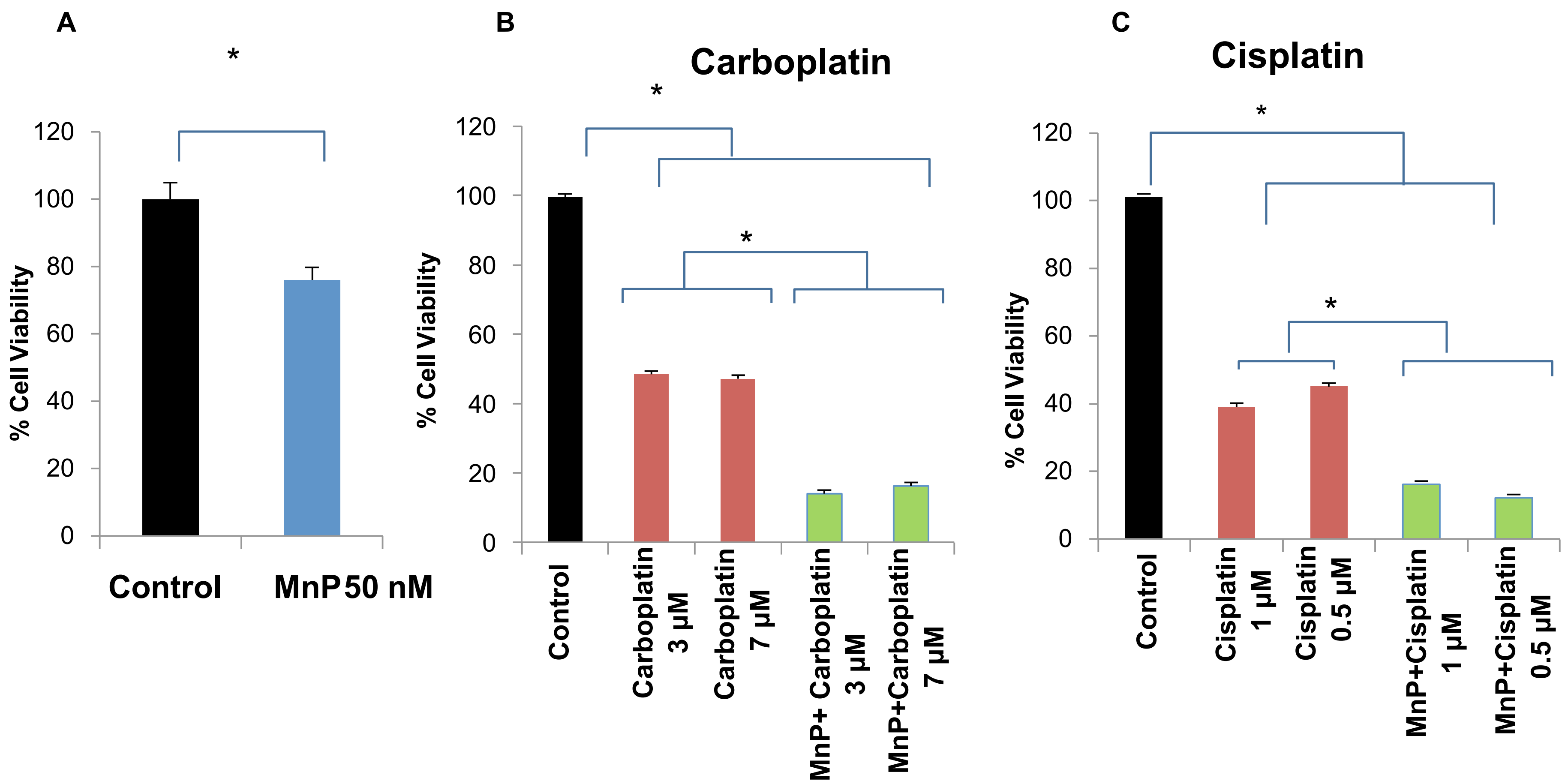

Supplementary Figure 2. Synergistic effect of MnP and Platinum compounds on TOV 21G. Colony survival assay was performed after 24 hr treatment. **(A)** MnP. **(B)** Carboplatin. **(C)** Cisplatin. MnP and drugs were added at the same time.

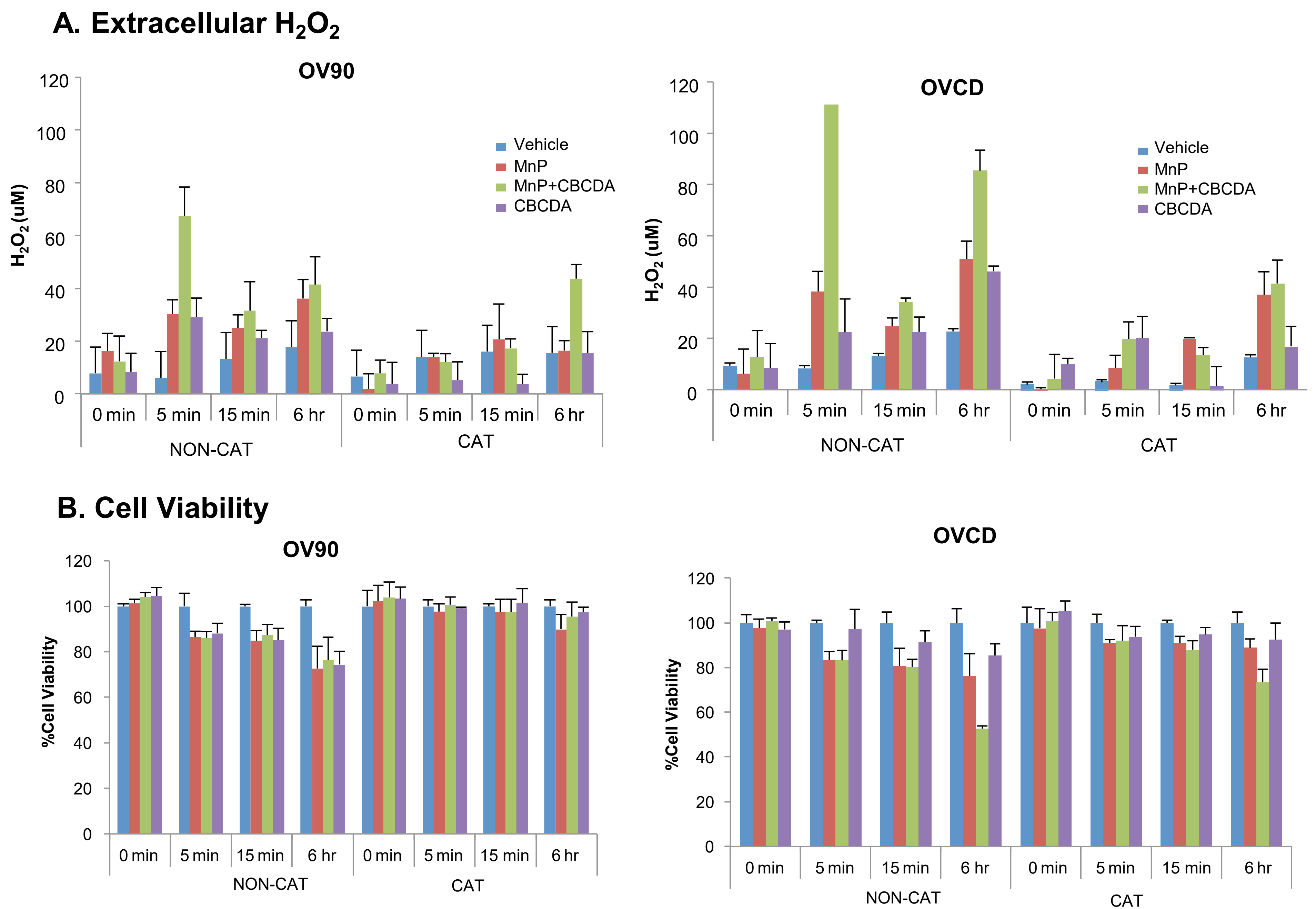

Supplementary Figure 3. Cell viability correlated with H_2O_2 production after MnP and CBCDA treatment at various time point. **(A)** Extracellular H_2O_2 treatment. **(B)** Cell viability with MTT.

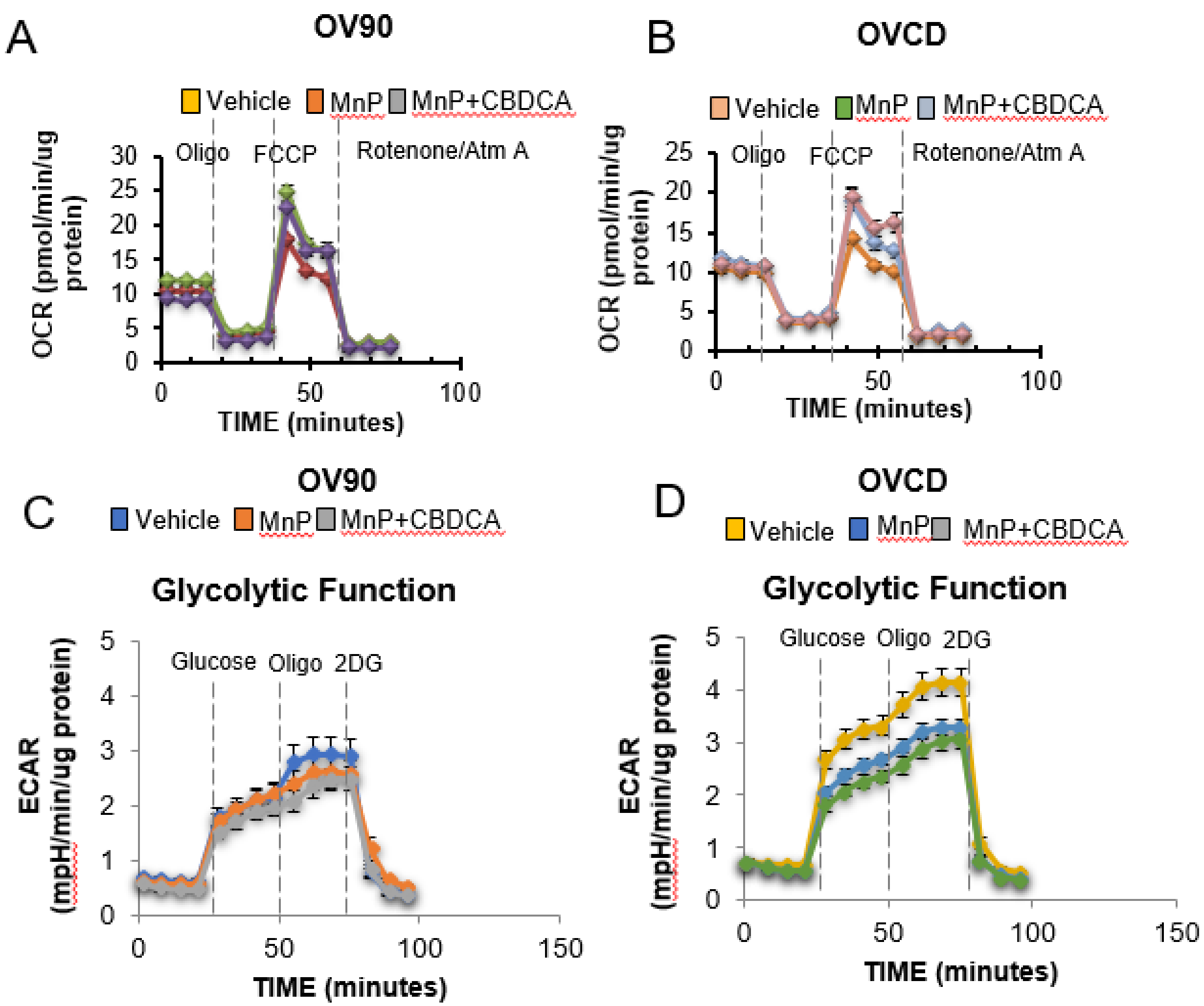

Supplementary Figure 4. Co-treatment of MnP and Carboplatin inhibited mitochondrial respiratory and ATP production in chemo-resistant ovarian cancer. Cells were treated with MnP and/or CBDCA for 6 hr. Live cells were then used for the measurement. Seahorse XF Analyzers was used to measure OCR and ECAR in OV90 **(A,C)** and in OVCD **(B,D)**. Oligo=Oligomycin. FCCP = Carbonyl cyanide-p-trifluoromethoxyphenylhydrazone. AtmA = Antimycin A. 2DG=2-deoxy-d-glucoses.

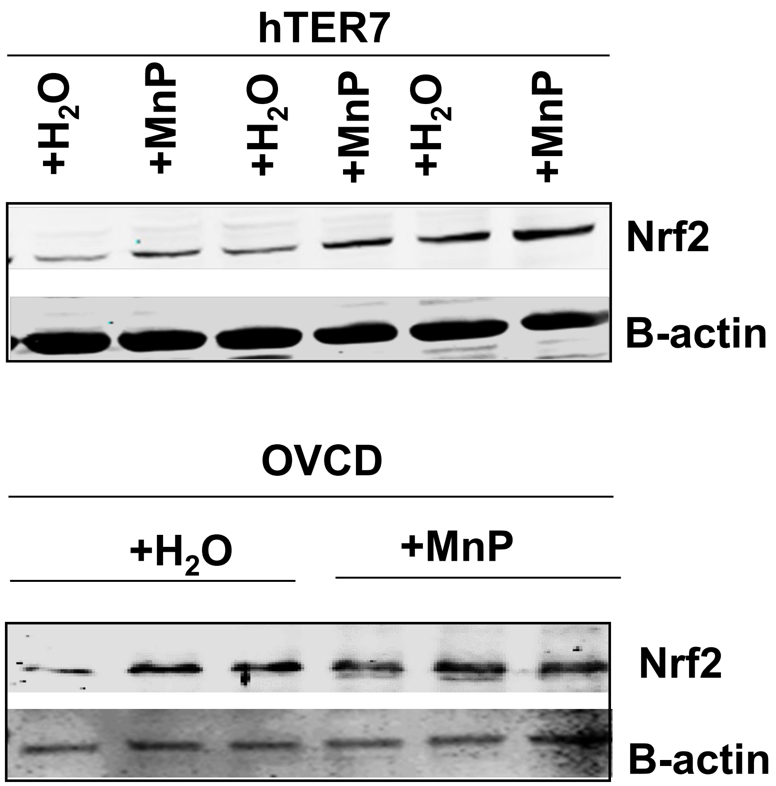

Supplementary Figure 5. Western blots demonstrated expression of Nrf2 protein after MnP treatment 6 hr.
